# Prevalence of Myopia Among Schoolchildren and the Impact of Increased Screen Time: A Systematic Review

**DOI:** 10.7759/cureus.66815

**Published:** 2024-08-13

**Authors:** Ahmed M Khalaf, Amro Y Alhazimi, Khalid K Almaymuni, Noura A Alsubaie

**Affiliations:** 1 General Practice, Imam Mohammad Ibn Saud Islamic University (IMSIU), Riyadh, SAU; 2 Ophthalmology, Imam Mohammad Ibn Saud Islamic University (IMSIU), Riyadh, SAU; 3 General Practice, King Fahad Specialist Hospital, Buraydah, SAU; 4 General Practice, King Saud Bin Abdulaziz University for Health Sciences, Riyadh, SAU

**Keywords:** systematic review, school children, screen time, myopia, prevalence

## Abstract

Myopia is the most frequent kind of refractive error and affects hundreds of millions of people. Growing evidence suggests that extended exposure to digital screens may exacerbate nearsightedness in children and young people. The purpose of this review is to compile data on the link between too much time in front of a screen and nearsightedness in young people so far. The principles of the Preferred Reporting Items for Systematic Reviews and Meta-Analyses (PRISMA) statement guided the methods used and the format of the resulting report. Articles published between January 1, 2000, and June 30, 2023, were found by searching Medline, PubMed, ScienceDirect, Google Scholar, CINAHL, the Cochrane Library, and Scopus. Studies reporting an association between myopia and time spent in front of screens in children and young adults were considered eligible. The initial search yielded a total of 1,251 studies. After eliminating duplicates and reviewing the titles and abstracts, 64 full-text articles were evaluated for eligibility. Ultimately, 15 of these studies were included in the final analysis. The 15 studies involved a total of 59,775 participants and were conducted in various countries, including China, Singapore, and the United States. Overall, the evidence did not support a significant association between screen time and myopia in school children and young adults. There is conflicting evidence on the link between screen time and myopia in children and adolescents. More research is needed to determine whether or not digital screen use is a risk factor for myopia. The complex association between screen time and myopia is not fully understood at this time because of the variability of the included studies. These results have significant public health implications since they may be used to guide recommendations for screen time use in children and the young population.

## Introduction and background

The most frequent refractive defect in children and young adults is myopia or near-sightedness [1]. As a result of parental ignorance and stigma, myopia has grown to be a worry. In addition, the COVID-19 epidemic has made the situation worse. Children's extended usage of digital displays has raised the prevalence of myopia and sped up its development. In fact, there has been a significant rise in the prevalence and incidence of myopia [2]. Compared to adults, children's axial lengths are shorter. The process of emmetropization begins as early as two years of life, evolves into myopia over time, and finally results in emmetropia by the age of 14. Hyperopia occurs when the eye's axial length is 18 mm at birth and reaches 23 mm by age 14, resulting in a 15 D myopic shift. This shift is counteracted by corneal flattening and lens thinning, leading to emmetropia [3]. Contrarily, myopia-prone children have a long axial length at birth, which overrides the emmetropization process and hastens the development of myopia in infancy. However, the growth slows down during adolescence and typically halts by the age of eighteen. In some individuals, myopia can continue to progress until they reach 25 years old. Any further progression after the age of 25 is usually due to lens thickening, which results in a myopic shift. Effective management of myopia requires proper classification [4].

Axial myopia, caused by an increase in the axial length of the eye, is the most commonly observed type of myopia in clinical settings. A myopic shift of three dimensions, often referred to as spherical myopia, is correlated with an increase in axial length of 1 mm. Meridian myopia, also referred to as myopic astigmatism, occurs when there are two focal points along two different axes. When the meridional discrepancy occurs along the vertical and horizontal axes, regular myopic astigmatism is identified. When the axis is not at 90 degrees or 180 degrees, oblique astigmatism develops [5]. The main cause of meridional myopia is the corneal curvature, not the globe's axial length. The third most essential element in the myopia classification is lenticular myopia. With time, the crystalline lens experiences significant changes that eventually affect refractive error. Therefore, it is important to check the lenticular component in adults for any advancement of myopia. Myopia can be classified as mild (differences between 0.5 and 4 D), moderate (differences between 4 and 8 D), or severe (differences more than 8 D) [5]. Refractive errors are suspected as a result of parents' concerns that their children read books close to their faces, frequently make mistakes while taking notes in class, cannot watch television from more than three feet away, and experience regular headaches [5].

Adolescent kids express definite complaints about having poor distance vision. It is important to determine whether refractive error is to blame for headache and eye discomfort complaints. Asthenopia, which presents as headaches, nocturnal alterations in vision, sporadic diplopia, and neck pain, is a complaint among astigmatic patients. In clinical practice, frequent squinting or globe deviation with loss of near vision is a common symptom [6]. Making a history helps with effective management. It is crucial to find out whether there is a family history of myopia, keratoconus, and retinal issues [7]. Myopic patients who have a family history of keratoconus and retinal issues should be examined for retinal and corneal conditions. For the diagnosis of lenticular myopia or ciliary spasm with increased intraocular pressure, a medical history of steroid treatment for allergic conjunctivitis in children is essential [8]. This is a current problem since it has been suggested that myopia development may be influenced by computers, smartphones, and tablets in recent years. The individual link between digital screen time and myopia risk has been investigated in several studies. Consistent proof that screen usage contributes to the development of myopia is missing, though [9]. Even before the advent of digital gadgets, education and close work had a significant impact on the growth in myopia rates. While screen time cannot account for the myopia epidemic in East Asian countries such as Singapore, Taiwan, Korea, and Japan - since the increase in myopia prevalence occurred decades before the invention of screen devices - it remains important to examine the secondary role of screen time [10]. However, the recent surge in screen time use may contribute further to the high prevalence of myopia in Asia and globally, and this latest trend warrants evaluation [11]. The goal of this systematic review is to assess the impact of increased screen time on myopia in school children.

## Review

Materials and methods

This study was conducted and reported in accordance with the Preferred Reporting Items for Systematic Reviews and Meta-Analyses (PRISMA) statement. A comprehensive search was conducted in several electronic databases, including Medline, PubMed, ScienceDirect, Google Scholar, CINAHL, the Cochrane Library, and Scopus for articles published between 1 January 2000 and 30 June 2023. The search strategy included the following keywords: "Myopia," "school children," "young adults," and "screen time." The search was limited to studies published in English.

Inclusion criteria for this review included studies that reported on the relationship between screen time and myopia in children and young adults, with a focus on axial length (AL) variations, myopia prevalence, myopia development, and usage of computers, video games, and mobile devices. The review included cohort, case-control, cross-sectional, and intervention trial studies, but excluded case reports and studies that did not report on the relationship between screen time and myopia.

Two researchers (AK and KA) independently reviewed the titles and abstracts of the identified articles to assess their relevance to the review. Full-text articles of potentially eligible studies were reviewed based on the inclusion criteria. Any discrepancies between the two investigators were resolved by a third investigator (NA).

Data extraction was performed independently by two investigators (AK and AA) using a standardized data extraction form. The extracted data included study characteristics, population characteristics, exposure and outcome measures, and effect estimates with their respective 95% confidence intervals (CIs). Any disagreements in data extraction were resolved by consensus or consultation with a third investigator (NA).

Results

**Figure 1:**
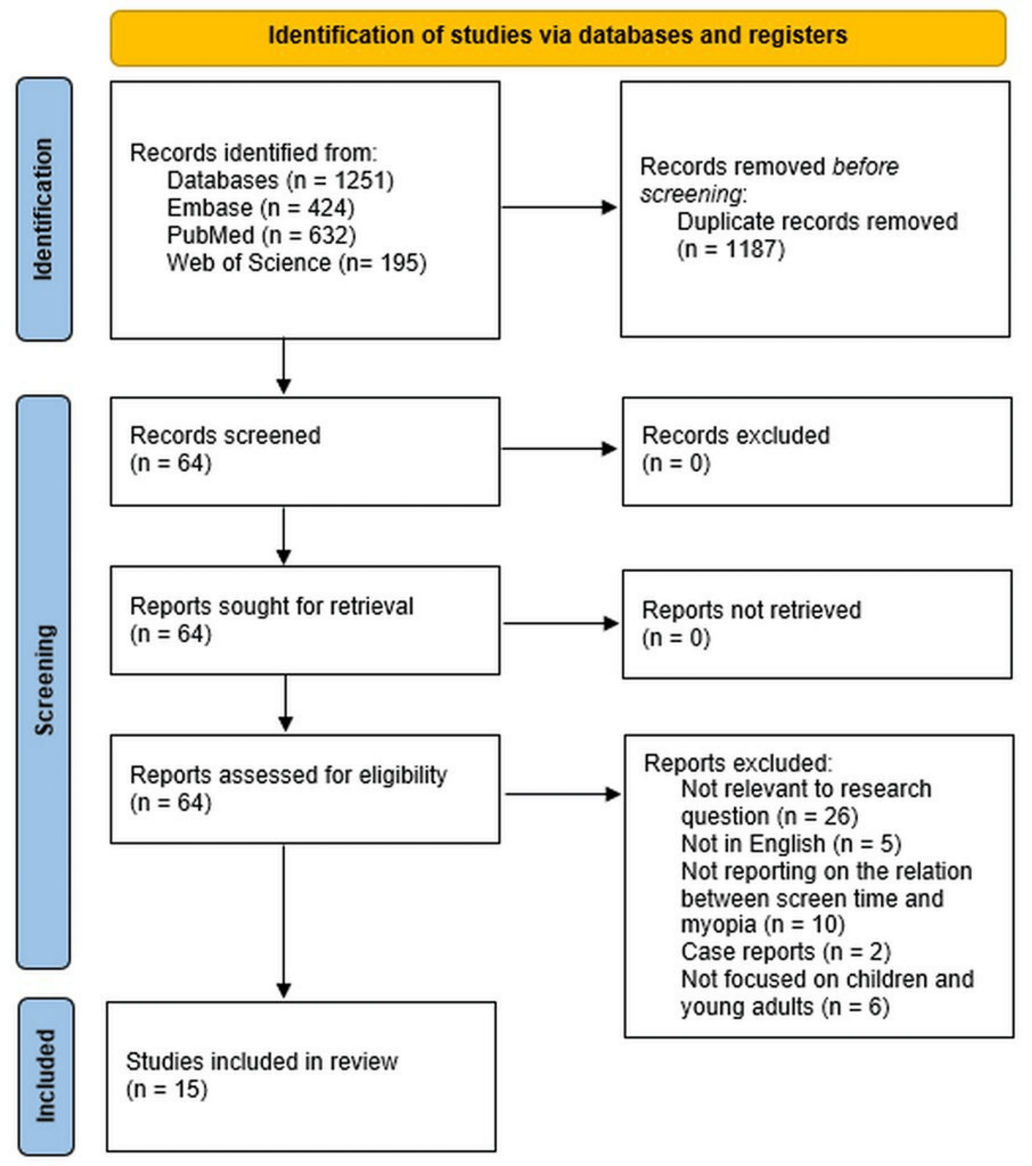
The PRISMA figures showing the steps to choose the studies for systematic review. PRISMA: Preferred Reporting Items for Systematic Reviews and Meta-Analysis, n: number

The initial search resulted in 1,251 studies. After eliminating duplicates and reviewing the titles and abstracts, 64 full-text articles were evaluated for eligibility. Out of these, 15 studies were included in the final analysis. Reasons for exclusion of studies included: irrelevant to the research question (n=26), not in English (n=5), not reporting on the relationship between screen time and myopia (n=10), case reports (n=2), and not focused on children and young adults (n=6) (Figure 1). The 15 studies that met the inclusion criteria were published between 2009 and 2021 and involved a total of 59,775 participants.

Table 1 includes 15 studies that examine the relationship between screen time and myopia. These studies varied in their design, location, and follow-up period. Most studies were cross-sectional [12-20], and the remaining were cohort studies [21-26]. All studies used a questionnaire to assess screen time, either for children or their families. Some studies did not report the response rate for their questionnaires, while others reported rates ranging from 27% to 98%. The studies included in Table 1 were conducted in various locations around the world, including China, India, Vietnam, the United States, and Australia. Seven studies were conducted in Asia [13-18,20], three in North America [19,21,23], and one in Australia [12]. The locations varied from rural areas to urban areas and suburban areas. The ethnicity of the study participants varied across the studies. Some studies included mainly Chinese participants, such as Lu et al., Qian et al., and Li et al. [13,17,25]. Other studies included mainly White participants, such as Ip et al. and Jones et al. [12,21]. Some studies included participants of mixed ethnicities, such as Jones-Jordan et al. and Guan et al. [18,23]. Finally, some studies included mainly Indian participants, such as Saxena et al. [15,26].

**Table 1:** Summary of studies investigating the relationship between screen time and myopia. NA: Not available, N: number

Authors	Year of publication	Study design	Location	Follow-up (year)	N of the sample	Ethnicity	Type of measure of screen time	Response rate
Ip et al. [12]	2008	Cross-sectional, population based	Sydney, Australia	NA	2353	Mainly White	Questionnaire	75%
Lu et al. [13]	2009	Cross-sectional	Xichang, rural China	NA	998	Chinese	Questionnaire	81%
Paudel et al. [14]	2014	Cross-sectional	Ba Ria, Vung Tau Province, Vietnam	NA	2238	Vietnamese	Questionnaire	NA
Saxena et al. [15]	2015	Cross-sectional	Delhi, India	NA	9884	Indian	Questionnaire	98%
Chua et al. [16]	2015	Cross-sectional analysis of data from a birth cohort	Singapore	NA	572	Mainly Chinese	Questionnaire	46%
Qian et al. [17]	2016	Cross-sectional	Mangshi Town, Yunnan province, rural China	NA	7681	Chinese	Questionnaire	93%
Guan et al. [18]	2019	Cross-sectional analysis of data from a randomized trial	Northwest of China	NA	19934	Chinese	Questionnaire	NA
Mutti et al. [19]	2002	Cross-sectional analysis of data from a cohort	Orinda, California, USA	NA	366	Mainly White	Questionnaire	NA
Saw et al. [20]	2002	Cross-sectional analysis of data from a cohort	Singapore	NA	1005	Mainly Chinese	Questionnaire	NA
Jones et al. [21]	2007	Cohort	Orinda, California, USA	12	514	Mainly White	Questionnaire	50%
Jones-Jordan et al. [22]	2012	Cohort	USA	20	835	Mixed ethnicities	Questionnaire	NA
Jones-Jordan et al. [23]	2011	Cohort	USA	16	1318	Mixed ethnicities	Questionnaire	27%
Wu et al. [24]	2013	Cohort, Interventional, School based	Suburban area of Southern Taiwan	1	571	Chinese	Questionnaire	NA
Li et al. [25]	2015	Cohort, School based	Urban areas of Anyang, Henan Province, Central China	2	1890	Chinese	Questionnaire	83%
Saxena et al. [26]	2017	Cohort, School based	Delhi, North India	1	9616	Indian	Questionnaire	97%

Table 2 summarizes the results of studies investigating the relationship between screen time and myopia. The table includes information on the authors and year of publication, age of participants, myopia definition, type of measure, screen time for near, and results. The studies in Table 2 were conducted in various locations around the world, including China, the United States, Singapore, and Australia. The ages of participants ranged from 3 to 16 years old. The myopia definition varied across studies, but most studies used cycloplegic autorefraction to measure refractive error. The type of measure used to assess screen time varied across studies, with some studies asking participants about their computer use, video game play, or handheld device use, while others simply recorded the number of hours spent on screens per week. Most studies defined myopia as SE ≤ -0.5 D, while four studies used a different definition of SE ≤ -0.75 D [19,21,23]. The results of the studies also varied, with some studies finding a significant positive association between screen time and myopia, while others found no significant association.

**Table 2:** Results of studies investigating the relationship between screen time and myopia. Data are expressed as mean ± S.D. or mean (SE). p-value < 0.05 denotes statistical significance. M = myopia group, Non-M = non-myopia group, OR = odds ratio, HM = high myopia, NS = not significant, S.D. = standard deviation, VA = visual acuity

Authors	Year of publication	Age	Myopia definition (SE)	Type of measure	Screen time for near	Results
Ip et al., [12]	2008	12-13	−0.5 D	Cycloplegic autorefraction	Computer use/playing handheld console games	Adjusted Mean spherical equivalent refraction (SER) per category, p> 0.05
Lu et al. [13]	2009	10-19	−0.5 D	Cycloplegic autorefraction	Playing video games/computer use	Mean ± S.D. (h/week) M = 6.2 ± 7.1; Non-M = 7.6 ± 7.7; p = 0.02 Mean ± S.D. (diopter-h/week) M = 18.9 ± 24.9; Non-M = 21.8 ± 24.7; p = 0.11
Paudel et al. [14]	2014	12-15	−0.5 D	Cycloplegic autorefraction if VA ≤ 6/12 + subjective refraction	Use of computers	Mean ± S.D. (h/week) M = 4.9 ± 6.5; Non-M = 4.3 ± 6.4; p = 0.077 OR = 1.02, 95% CI 1.00, 1.04; p = 0.022
Saxena et al. [15]	2015	5-15	−0.5 D	Cycloplegic autorefraction + Retinoscopy if VA	Using computers/video and playing mobile games	OR = 4.5, 95% CI 2.33, 8.98; p < 0.001 for 1-4 h/week, OR = 8.1, 95% CI 4.05, 16.2; p < 0.001 for >4 h/week
Chua et al. [16]	2015	3	−0.5 D	Cycloplegic autorefraction	Using computers/playing with handheld devices	OR = 1.04, 95% CI 0.67, 1.61; p = 0.86/ OR = 0.92, 95% CI 0.31, 2.74; p = 0.88
Qian et al. [17]	2016	5-16	−0.5 D HM ≤ −6.0 D	Cycloplegic autorefraction	Computer use	OR (M) = 1.17, 95% CI 1.03, 1.32; p = 0.015 OR (HM) = 2.31, 95% CI 1.17, 4.57; p = 0.016
Guan et al., [^18^]	2019	10.6	−0.5 D	Cycloplegic autorefraction if VA ≤ 6/12 + subjective refinement	Computer/smartphone use	Adjusted Mean SER per category, p> 0.05
Mutti et al. [19]	2002	13-14	−0.75 D	Cycloplegic autorefraction	Playing video games or working on a computer at home	Mean ± S.D. (h/week) M = 2.7 ± 4.1; Emmetropia = 2.2 ± 3.2; p = NS
Saw et al., [20]	2002	7-9	−0.5 D	Cycloplegic autorefraction	Computer use (Yes, No)	Low M = yes, 24.4; no, 24.3, HM = yes, 10.0; no, 5.4, Non-M = yes, 65.6; no, 70.3, p = 0.03 for HM versus Non-M
Jones et al. [21]	2007	8-9	−0.75 D	Cycloplegic autorefraction	Plays video games/uses a computer	Mean ± S.D. (h/week) M = 2.52 ± 2.92; Non-M = 2.45 ± 2.81 OR = 1.01, 95% CI 0.94, 1.09; p = NA
Jones-Jordan et al. [22]	2012	6-14	0.75D	Cycloplegic autorefraction	Plays video games/uses a computer (h/week)	Adjusted β = −0.02, 95% CI −0.08, 0.03 before and after progression [β = −0.02, 95% CI −0.08, 0.03]
Jones-Jordan et al., [23]	2011	6-14	−0.75D	Cycloplegic autorefraction	Computer/playing video games	Adjusted Least-Squares Mean Difference between Became-Myopic Hours and Estimated Emmetropic Hours: p < 0.05 at onset and years 1, 2, 3 & 5
Wu et al., [24]	2013	7-11	−0.5 D	Cycloplegic autorefraction	Playing on a computer	Adjusted myopic shift for non-myopic 0.10, 95% CI −0.08, 0.27; p = 0.279 and myopic −0.08, 95% CI −0.27, 0.12; p = 0.453
Li et al., [25]	2015	10-15	−0.5 D	Cycloplegic autorefraction	Plays video games/uses a computer (h/day)	Adjusted β for middle tertile and high tertile: all p> 0.05
Saxena et al., [26]	2017	5-15	−0.5 D	Cycloplegic autorefraction + Retinoscopy	Plays video games/uses a computer (h/week)	OR = 1.89, 95% CI 1,42, 2.49; p < 0.001 for 4-7 h/week, OR = 3.53, 95% CI 2.51, 4.95; p < 0.001 for >7 h/week

Studies Reporting a Significant Positive Association

Several studies in Table 2 found a significant positive association between screen time and myopia. Saxena et al. [15] and Saxena et al. [26] both reported that increased computer and video game use was associated with higher odds of myopia [15,26]. Similarly, Qian et al. found that computer use was associated with increased odds of myopia, particularly in participants with high myopia where the odds ratio for computer use was 1.17 for all myopic participants and 2.31 for those with high myopia [17]. Lu et al. reported that playing video games and using computers was associated with myopia [13], and Paudel et al. found a small but statistically significant association between computer use and myopia [14]. Finally, Saw et al. found a significant association between computer use and myopia only in participants who were not emmetropic [20]. For example, Saxena et al. found that participants who used computers and played video and mobile games for more than four hours per week had higher odds of myopia than those who used screens less often [15], while Saxena et al. reported that participants who used computers and played video games for more than seven hours per week had higher odds of myopia than those who used screens less often [26].

Studies Reporting No Significant Association

Several studies in Table 2 reported no significant association between screen time and myopia. Chua et al. found no significant association between handheld device use or computer use and myopia [16], and Guan et al. also found no significant association between screen time and myopia [18]. Mutti et al., Jones et al., Ip et al., Jones-Jordan et al., and Wu et al. all reported no significant association between screen time and myopia [12,19,21,22,24].

Studies Reporting a Significant Association with Myopia Progression

Two of the studies in Table 2 reported a significant association between screen time and myopia progression. Jones-Jordan et al. found that participants who became myopic spent more hours using computers than those who remained emmetropic [23]. Additionally, Qian et al. reported that computer use was associated with increased odds of myopia progression [17].

Discussion

Myopia, often known as nearsightedness, is an increasingly prevalent refractive defect [27]. Myopia is on the rise, and scientists have identified a number of causes, including genetics, environment, and lifestyle changes (such as more screen usage) [28,29]. The results of research examining the link between screen time and myopia have been mixed, with some finding a favorable effect and others finding none.

Myopia and screen time is a controversial topic with conflicting research. Some studies have identified a positive correlation between screen usage and nearsightedness, while others have found either no correlation or even a negative correlation. Our research found that the literature evaluation also included a number of contradictory findings and screen time increased significantly compared to before the pandemic, according to a cohort study of 1793 Hong Kong schoolchildren studying its effects [30]. Screen usage is positively associated with the development of myopia, according to the results of two more observational studies [31,32]. The advantages of digital technology today are unquestionable. Myopia is on the rise in elementary and secondary school pupils due, in part, to their growing use of electronic devices [33-36]. After controlling for factors including time spent outside and close work, He et al. indicated that screen usage was independently connected with myopia risk [37]. Myopia was found to be linked to time spent indoors and outdoors by Xiong et al. [38]. They hypothesized that one strategy for warding off myopia was to spend less time in front of a screen and more time in the great outdoors [38]. Screen time was found to be significantly linked with myopia in a meta-analysis and systematic review conducted by Wang et al.. They hypothesized that indoor activities and less time spent outside could mitigate the link [39].

Reducing outdoor activity, which in turn reduces exposure to natural light and time spent looking at distant objects, could be one explanation for the negative effect of screen time on myopia, while the exact processes through which screen time may affect myopia are yet unclear. Dirani et al. suggested that the increasing use of electronic devices may be to blame for people not getting enough exercise [40]. However, there is little evidence to support the idea that children's time spent outdoors is anything more than a substitute for increased reliance on electronic media [41,42]. The use of electronic games or mobile devices, especially late at night, is also thought to contribute to the rise in myopia. Myopia risk factors have previously included exposure to blue light at night and poor sleep quality [43]. Grzybowski et al., Qu et al., and Shneor et al. all suggested that the use of LED lights for schoolwork, low-light environments, and sleep had emerged as new study considerations [44-46]. However, there was insufficient evidence linking sleep disorders to the onset of myopia in teenagers [47]. Multiple explanations have been presented for the link between sleep deprivation and myopia, but the underlying mechanism remains unknown.

Outdoor activity and the influence of screen time, a potential substitute for conventional print-based literacy, continue to be the most significant controllable factors contributing to myopia [48]. Recent research suggests that educational screen time may also be replacing reading and writing (e.g., computer or video games). Children use smartphones primarily for entertainment (49%) and education (19%) [49]. Time spent in front of a screen may not be a direct cause, but it could be a substitute for other forms of near labor. Myopia has been associated with a combination of close-up work and insufficient outdoor time in children [40]. A significant proportion of children aged 6-15 years old (48%) engage in indoor activities such as video gaming and electronic media consumption, potentially contributing to this issue [50]. These findings could indicate a replacement effect between outdoor and digital screen time, with the latter serving as a surrogate for indoor activity [51]. Studies yet have not yielded enough information to isolate the unique effects of children's screen time relative to other non-screen activities. There has to be more research done in this area.

Our results highlight the need to conduct studies in which objective assessments of screen time can be collected to eliminate recollection bias, such as through the deployment of applications that monitor usage on participants' mobile devices. Screen time was measured in all included research using parental or child self-report through questionnaires. Due to the possibility of erroneous reporting or recollection bias on the part of participants, the questionnaires have not been validated against external objective metrics.

## Conclusions

There is conflicting data on whether or not children and adolescents who spend long periods of time in front of digital screens are more likely to develop myopia. To begin, the emergence of mobile devices in the previous decades has led to an increase in digital screen time. Given the contradictory findings, more research into the link between screen usage and myopia is warranted. In metropolitan Asia, the prevalence of myopia rose mostly with increasing education a few decades ago, rather than recently along with an increase in screen usage. Second, no apparent correlation exists between screen time and near-work hours, suggesting that traditional academic reading and writing may be replaced by digital screen activities, while recreational screen use might be more restricted. It is crucial to know whether or not myopia and the progression of myopia in myopic and high myopic populations are exacerbated by exposure to digital screen devices. There needs to be more research utilizing objective screen time measures to understand why kids are spending more and more time in front of devices.
